# Identification of key lipid metabolism-related genes in kidney fibrosis: implications for chronic kidney disease management

**DOI:** 10.3389/fphys.2025.1652513

**Published:** 2025-09-19

**Authors:** Qiuyu Cao, Longhui Liu, Sai Zhou, Yang Fei, Yi Guo, Yin Li, Shengyun Sun, Aicheng Yang

**Affiliations:** ^1^ Department of Nephrology, Affiliated Jiangmen TCM Hospital of Jinan University, Jiangmen, Guangdong, China; ^2^ Integrated Chinese and Western Medicine Postdoctoral Research Station Jinan University, Guangzhou, Guangdong, China; ^3^ Department of Gynecology, Affiliated Jiangmen TCM Hospital of Jinan University, Jiangmen, Guangdong, China; ^4^ Department of Gynecology, Zhejiang Chinese Medical University, Zhejiang, China; ^5^ Department of Gynecology, The Third Affiliated Hospital of Zhengzhou University, Zhengzhou, Henan, China; ^6^ Department of Nephrology, The First Affiliated Hospital of Jinan University, Guangzhou, Guangdong, China

**Keywords:** kidney fibrosis, lipid metabolism, machine learning, immune infiltration, chronic kidney disease

## Abstract

**Background:**

Kidney fibrosis (KF) represents a critical pathological alteration in the end stage of chronic kidney disease (CKD) and is the ultimate cause of mortality. Lipid metabolism plays a significant role in the pathogenesis of KF. Therefore, biomarkers associated with lipid metabolism will be identified to guide the treatment and management of CKD.

**Methods:**

Three datasets obtained from the GEO database, along with 760 lipid metabolism-related genes sourced from two databases, were utilized to identify lipid metabolism-associated differentially expressed genes (LMDEGs) in KF. Subsequently, we performed GO, KEGG and ssGSEA enrichment analysis to elucidate the characteristics of LMDEGs. Then, machine learning was applied to identify core LMDEGs, Least Absolute Shrinkage and Selection Operator (LASSO) was utilized to construct a diagnostic model, and Receiver Operation Curve (ROC) was operated to evaluate the diagnostic performance. We used unsupervised hierarchical clustering to identify subtypes of KF associated with lipid metabolism and employed Gene Set Variation Analysis (GSVA) to examine differences among clusters. Finally, transcription factor and miRNA regulatory networks upstream of core LMDEGs were constructed using Cytoscape software.

**Results:**

We identified 54 LMDEGs and constructed a six core LMDEGs (UGCG, SFRP1A6, OSBPL6, INPP5J, PNPLA3, and GK) predictive model by LASSO regression, achieving area under the curve (AUC) values ranging from 0.723 to 0.774. ssGSEA confirmed that these six core LMDEGs exhibited significant positive or negative correlations with immune cell infiltration. Based on the expression profiles of these core LMDEGs, KF samples were categorized into three distinct subtypes. One subtype is predominantly characterized by enhanced lipid and energy metabolism, another exhibits features of inflammation and immune response activation, while the third displays an intermediate pattern between the two extremes. Moreover, the regulatory network of these core LMDEGs shared several common transcription factors, suggesting a potential interplay between lipid metabolism and immune responses in the pathogenesis of KF.

**Conclusion:**

We have identified six core LMDEGs that are significantly associated with KF. Based on this, we have established three distinct clusters related to lipid metabolism in KF, which may provide valuable insights into the treatment and management of CKD.

## 1 Introduction

Chronic kidney disease (CKD) is defined as the presence of structural or functional abnormalities in the kidneys persisting for more than 3 months, resulting in adverse effects on overall health. Clinically, CKD is diagnosed when the estimated glomerular filtration rate (eGFR) falls below 60 mL/min/1.73 m^2^ or when the albumin-to-creatinine ratio (ACR) reaches or exceeds 30 mg/g. Current data indicate that the global prevalence of CKD is estimated to range from 10% to 14% (Chen et al., 2019). However, due to the asymptomatic nature of early-stage CKD, the true prevalence may be substantially underestimated. Irrespective of etiology, CKD progresses through a series of molecular mechanisms including apoptosis, chronic inflammation, oxidative stress, fibrosis, and metabolic disturbances, culminating in irreversible loss of nephrons, microvascular damage, end-stage renal disease (ESRD), and premature mortality (GBD Chronic Kidney Disease Collaboration, 2020; Ruiz-Ortega et al., 2020). Projections indicate that by 2040, CKD will rank as the fifth leading cause of death globally (Foreman et al., 2018), posing a significant challenge to public health systems and necessitating heightened awareness and substantial investment in research (Francis et al., 2024; Jadoul et al., 2024).

KF represents a ubiquitous pathological progression and ultimate manifestation of chronic kidney disease, characterized by glomerular sclerosis, tubular atrophy, chronic interstitial inflammation, fibrogenesis, and vascular rarefaction (Yuan et al., 2022). Researches has elucidated that the pathogenesis of renal fibrosis is multifaceted, involving interactions among various cell types including mesenchymal cells, immune cells, and specific tubular epithelial cells (Li et al., 2022; Kuppe et al., 2021), as well as multiple signaling pathways such as Notch, Wnt, and Hedgehog (Huang et al., 2023). Moreover, with advancements in epigenetic studies, the mechanisms underlying the interaction between aberrant gene expression and environmental changes have been progressively uncovered, emerging as a novel focus for the prevention, diagnosis, and therapeutic targets of renal fibrosis (Feinberg, 2018). Despite some anti-fibrotic agents (Huang et al., 2023), including RAS blockers, SGLT2 inhibitors, vasopressin receptor 2 antagonists, and non-steroidal anti-mineralocorticoids, being capable of delaying the progression of chronic kidney disease to a certain extent, they are unable to reverse the established pathological outcomes of renal fibrosis.

Lipid metabolism encompasses a series of biochemical processes, including synthesis, degradation, transport, and storage of lipids within the body. These processes are crucial for maintaining cell membrane integrity, energy storage, and signal transduction. Disruptions in lipid metabolism have been implicated in the pathogenesis and progression of various diseases, such as cancer (Terry and Hay, 2024), cardiovascular and cerebrovascular disorders (Sakers et al., 2022), and non-alcoholic fatty liver disease (Scorle et al., 2022). Research has demonstrated that excessive lipid accumulation and lipid-induced toxicity are frequently associated with CKD and fibrosis (Chen et al., 2022). This pathological process can be effectively mitigated by enhancing fatty acid oxidation in renal tubular epithelial cells or inhibiting fatty acid transporters (Li et al., 2025; Chen et al., 2020). Consequently, an in-depth investigation into the specific mechanisms of lipid metabolism in KF and the exploration of potential intervention targets could facilitate the development of novel therapeutic strategies, ultimately benefiting patients with CKD. However, current research predominantly focuses on analyzing differences in lipid metabolism markers in KF or examining it as a complication of other diseases, while studies targeting lipid metabolism pathways specifically for diagnosing or treating KF remain relatively limited.

Based on this, we employed bioinformatics tools to identify potential targets associated with lipid metabolism that influence the occurrence and progression of KF. Initially, three transcriptome datasets were retrieved from the GEO database for analysis. After identifying DEGs using sva, we intersected these DEGs with a previously reported set of 760 lipid metabolism-related genes to obtain LMDEGs. Subsequently, GO functional analysis and KEGG pathway enrichment analysis were performed on the LMDEGs. Importantly, through various machine learning algorithms, we identified six biomolecules with diagnostic significance: UGCG, SERPINA6, OSBPL6, INPP5J, PNPLA3, and GK, which were subsequently validated. To further elucidate the role of these six core lipid metabolism-related genes in KF development, we conducted immune infiltration analysis, gene correlation analysis, REACTOME pathway enrichment analysis, and constructed their upstream regulatory networks. Additionally, based on these six core genes, unsupervised clustering was performed using ConsensusClusterPlus on all samples, resulting in three distinct clusters, for which pathway scores were calculated. In conclusion, our study has revealed lipid metabolism genes implicated in the progression of renal fibrosis, which may serve as potential targets for guiding clinical diagnosis and treatment.

## 2 Methods

### 2.1 Data sources and data processing methodologies

Raw gene expression data for patients with kidney fibrosis were obtained from three datasets (GSE76882, GSE22459, and GSE65326) available in the GEO database (www.ncbi.nlm.nih.gov/geo/). The GSE76882 dataset, collected using the Affymetrix HT HG-U133+ PM Array Plate (GPL13158), includes 175 samples from kidney fibrosis cases and 99 samples from normal kidney tissues (Modena et al., 2016). The GSE22459 dataset provides information on 40 kidney fibrosis and 25 healthy kidney RNA samples, utilizing the Affymetrix Human Genome U133 Plus 2.0 Array (GPL570) (Park et al., 2010). Lastly, the GSE65326 dataset, which employed the Illumina HumanHT-12 v4.0 Expression BeadChip (GPL10558), contains 16 kidney fibrosis and 6 normal samples, after excluding one sample due to incomplete data (Toki et al., 2014). In the machine learning model described subsequently, we selected GSE76882 as the training dataset, while the other two datasets were chosen for validation purposes.

### 2.2 Identification and screening of DEGs

Gene expression profiles were generated by normalizing the data and batch-correcting the expression values using the limma and sva packages in R. This normalization was performed after merging the three datasets, resulting in a consolidated dataset comprising 14,326 genes and 361 samples. Principal Component Analysis (PCA) was conducted using the FactoMineR and factoextra packages to visualize the adjustments through three-dimensional scatter plots. Following data homogenization, the Linear Models for Microarray Data (LIMMA) package was utilized to identify DEGs between the kidney fibrosis and control groups. To enhance the reliability of DEGs identification, probe sets with an adjusted p-value <0.05 and |logFC| > 0.5 were designated as significantly differentially expressed. The identified DEGs were visually represented using a volcano plot and a heatmap.

### 2.3 Functional analysis of DEGs and GSEA

To elucidate the biological functions of DEGs, we employed the ClusterProfiler package for comprehensive functional analyses. These analyses encompassed GO and KEGG pathway enrichment. GO annotations were categorized into three main aspects: biological process (BP), molecular function (MF), and cellular component (CC). To account for multiple testing, the Benjamini-Hochberg method was applied to adjust p-values, resulting in false discovery rate (FDR) corrections. A significance threshold of FDR <0.05 was established. Additionally, GSEA was conducted using the ClusterProfiler package to calculate the enrichment scores of pathways associated with the identified DEGs.

### 2.4 Identification and analysis of DEGs associated with lipid metabolism

We defined differentially expressed lipid metabolism-related genes as LMDEGs, which were identified by intersecting lipid metabolism-related genes with DEGs. Prior to this, lipid metabolism-related genes were sourced from two reputable databases: Reactome Metabolism of Lipids (http://www.gsea-msigdb.org/gsea/msigdb/human/geneset/REACTOME_METABOLISM_OF_LIPIDS) and WP lipid metabolism pathway (http://www.gsea-msigdb.org/gsea/msigdb/human/geneset/WE_LIPID_METABOLISM_PATHWAY). Subsequently, LMDEGs were subjected to GO and KEGG enrichment analyses as previously described. Finally, a hot plot and a heatmap were generated to illustrate the differential expression levels of LMDEGs between the KF and control groups. To further elucidate the characteristics of LMDEGs, a box plot was created using the ggplot2 package in R.

### 2.5 Identification and selection of core LMDEGs

After intersecting with the list of lipid metabolism-related genes, 54 candidate genes were subsequently analyzed using machine learning algorithms for feature selection. We integrated ten classical algorithms: Random Survival Forest (RSF), Least Absolute Shrinkage and Selection Operator (LASSO), Gradient Boosting Machine (GBM), Survival Support Vector Machine (Survival-SVM), Supervised Principal Components Analysis (SuperPC), Ridge Regression, Partial Least Squares Regression for Cox models (plsRcox), CoxBoost, Stepwise Cox, and Elastic Net (Enet). Notably, RSF, LASSO, CoxBoost, and Stepwise Cox possess dimensionality reduction and variable screening capabilities, which we combined with other algorithms to form various machine-learning algorithm ensembles. Based on the AUC metric, five top-performing machine learning algorithms—namely Stepglm [backward] + GBM, Stepglm [both] + GBM, LASSO + GBM, GBM, and Stepglm [backward] + RF—were selected to identify the core LMDEGs from a pool of 54 candidate genes. The diagnostic efficacy of these models was evaluated using ROC curves and their corresponding AUC values.

### 2.6 Comprehensive analysis of immune cell infiltration

ssGSEA was conducted to evaluate immune infiltration based on the expression profiles of 29 immunity-related signatures. The analyses encompassed the interrelationships among various types of immune cells, the differences in immune infiltration between fibrotic and healthy kidneys, as well as the correlations between immune cells and key LMDEGs.

### 2.7 Unsupervised hierarchical clustering analysis

The normalized expression microarray data for each patient were collected and subsequently analyzed using unsupervised hierarchical clustering via the ConsensusClusterPlus package in R.

### 2.8 Conducting pathway enrichment analysis

The HALLMARK, KEGG, and REACTOME pathways were retrieved from the MSigDB database to serve as the reference set. The GSVA scores for each pathway were computed using the ssGSEA function in the GSVA package within R. These GSVA scores represent the absolute enrichment levels of the respective gene sets. Subsequently, the GSVA scores were compared across two clusters utilizing the limma package.

### 2.9 Development and analysis of a regulatory network

Regulatory data pertaining to miRNAs and transcription factors were retrieved from the RegNetwork database (https://regnetworkweb.org/) for the upstream prediction of core LMDEGs. Subsequently, the regulatory network was constructed utilizing Cytoscape software.

### 2.10 Statistical analysis

All statistical analyses were conducted using R version 4.2.2. Heatmaps were generated utilizing the R package pheatmap. Lasso analysis was carried out with the R package glmnet. Box plots, lollipop plots, and volcano plots were created using the R package ggplot2.

## 3 Results

### 3.1 DEGs were identified and subjected to GO and KEGG enrichment analyses

**FIGURE 1:**
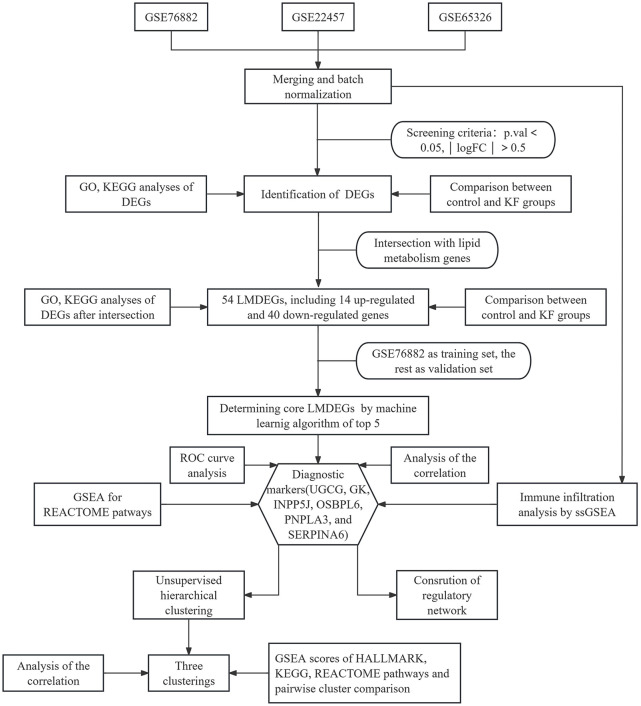
Flow chart of the study.

**FIGURE 2:**
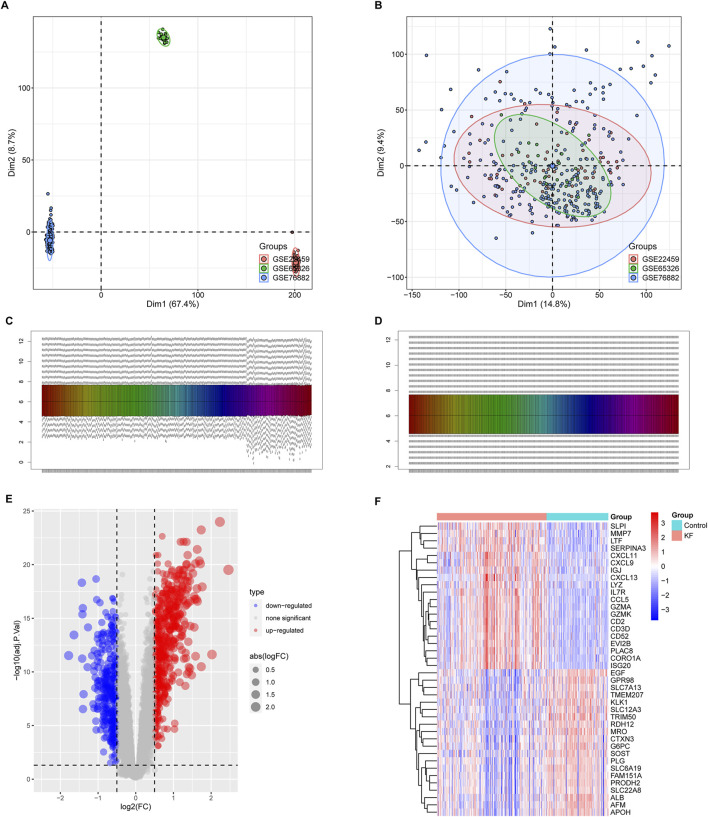
Identification and analysis of differentially expressed genes (DEGs) from the integrated expression profiles of the GSE76882, GSE22457, and GSE65326 datasets. Principal Component Analysis (PCA) plots showing 361 samples from aforementioned three databases prior to **(A)** and subsequent to **(B)** batch effect removal. Sample distribution prior to **(C)** and following **(D)** the homogenization of the datasets. **(E,F)** A volcano plot illustrating upregulated genes as red points and downregulated genes as blue points **(E)**. The heatmap visualizing clusters of genes with distinct expression patterns between the control group and kidney fibrosis samples **(F)**.

We have organized this study and its methodology as illustrated in Figure 1. The three kidney fibrosis datasets (GSE76882, GSE22459, GSE65326) were incorporated into the study and merged using the limma and sva algorithms to eliminate batch effects. Distribution patterns of the fibrotic cases, both before and after normalization, were visualized using PCA (Figures 2A,B) and box plots (Figures 2C,D). Following normalization, all samples were subjected to variance analysis using the limma package. Distinct gene expression patterns between healthy and fibrotic kidneys were identified based on the criteria of an adjusted p-value 0.05. We identified 943 DEGs, comprising 583 upregulated and 360 downregulated genes associated with KF, as illustrated in the volcano plot and heatmap (Figures 2E,F).

**FIGURE 3:**
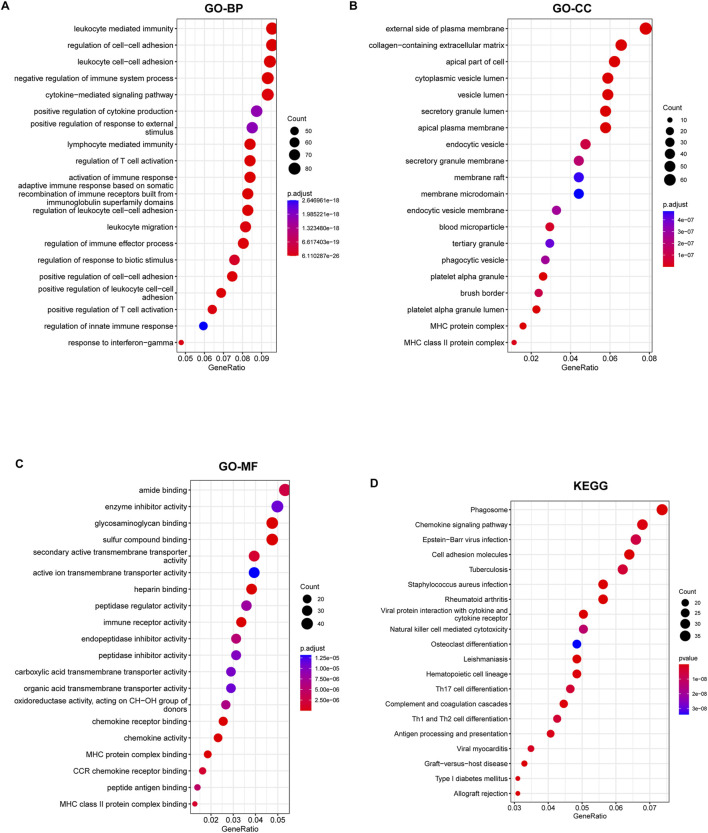
GO annotation and KEGG enrichment analyses of DEGs. **(A–C)** GO annotation of DEGs in association with annotated biological process (BP), cellular component (CC), and molecular function (MF). **(D)** Demonstration of KEGG enrichment analysis results. Pathways are ranked based on their GeneRatio, with the size bubbles indicating the number of enriched genes and the colors representing the p-values.

Subsequently, we conducted pathway enrichment analyses on the DEGs associated with KF. GO analysis indicated that these DEGs were significantly enriched in fibrotic processes, including the cytokine-mediated signaling pathway, collagen-containing extracellular matrix, and receptor-ligand activity (Figures 3A–C). Additionally, KEGG analysis underscored their involvement in the phagosome, chemokine signaling pathway, and cell adhesion molecules (Figure 3D). The enrichment of these pathways suggests that the DEGs are linked to chemokine signaling and the excessive production of extracellular matrix, both of which contribute to the progression of KF.

### 3.2 Lipid metabolism was intricately associated with the pathogenesis and progression of KF

**FIGURE 4:**
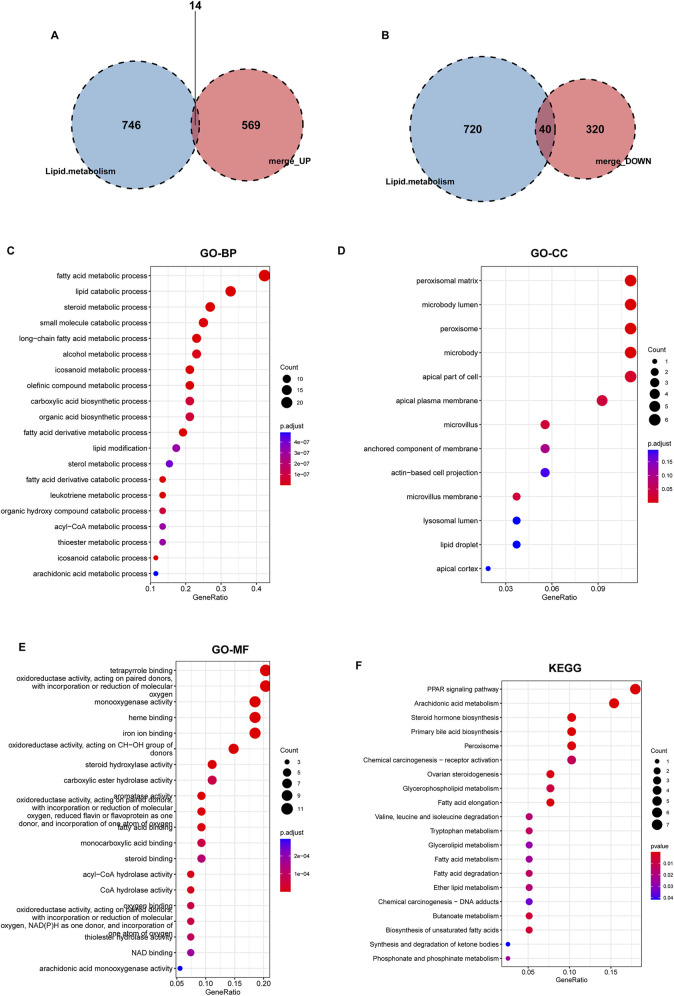
Identification of lipid metabolism-related genes from DEGs, followed by GO and KEGG enrichment analyses. Two Venn diagrams displaying the intersection of upregulated **(A)** and downregulated **(B)** DEGs with lipid metabolism-related genes derived from lipid metabolism datasets. **(C–F)** GO annotation, encompassing BP, CC, MF, and KEGG pathway enrichment analysis of 54 lipid metabolism-related DEGs that overlap with known lipid metabolism genes.

The 943 DEGs identified using the limma package were intersected with 760 lipid metabolism genes, resulting in the identification of 54 differential genes associated with lipid metabolism. This subset comprises 14 upregulated genes and 40 downregulated genes (Figures 4A,B). Subsequently, we conducted GO annotation and KEGG pathway enrichment analyses to explore the characteristics of these 54 LMDEGs. The results of the GO annotation analysis indicated that these genes are enriched in processes related to lipid metabolism and oxidative stress, including the fatty acid metabolic process, lipid catabolic process, steroid metabolism, peroxisomal matrix, peroxisome, microbody lumen, and the incorporation or reduction of molecular oxygen (Figures 4C–E). Furthermore, the KEGG analysis revealed that these genes are involved in the PPAR signaling pathway, arachidonic acid metabolism, steroid hormone biosynthesis, primary bile acid biosynthesis, and peroxisome pathways (Figure 4F). These findings suggested that lipid metabolism disorders and oxidative stress may represent significant pathological mechanisms contributing to the occurrence and progression of KF.

**FIGURE 5:**
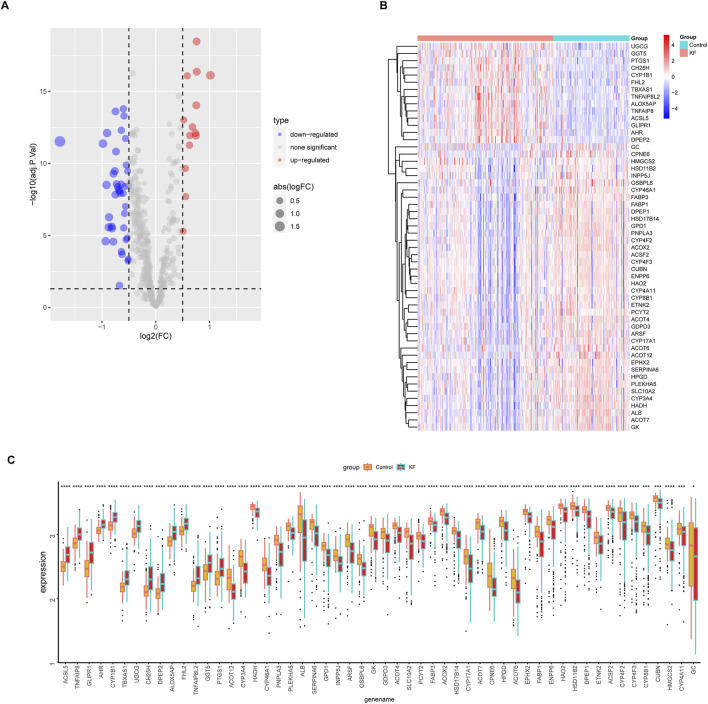
Differential expression profiles of 54 LMDEGs between control and kidney fibrosis groups. **(A)** A volcano plot showing upregulated genes as red points and downregulated genes as blue points. **(B,C)** A heatmap and a box plot visually displaying the expression patterns of 54 LMDEGs between healthy and fibrotic kidneys. ns denotes no significant difference, * denotes p < 0.05, ** denotes p < 0.01, *** denotes p < 0.001.

To further elucidate the expression levels of 54 LMDEGs in KF (Figure 5A), we compared the KF group to the control group. Our findings revealed that 14 genes, including UGCG, GGT5, PTGS1, CH25H, CYP1B1, FHL2, TBXAS1, TNFAIP8L2, ALOX5AP, TNFAIP8, and ACSL5, exhibited high expression levels in the kidney fibrosis group. Conversely, 40 genes, such as GC, CPNE6, HMGCS2, HSD11B2, INPP5J, OSBPL6, and CYP46A1, showed low expression levels in the same group (Figures 5B,C). These outcomes suggested that these genes may play a significant role in the progression of KF.

### 3.3 Identification and validation of core LMDEGs

To further investigate characteristic lipid metabolism regulators associated with kidney fibrosis, the 54 LMDEGs identified across three datasets were incorporated into our machine learning-based integrative model to establish a consensus KF signature.

**FIGURE 6:**
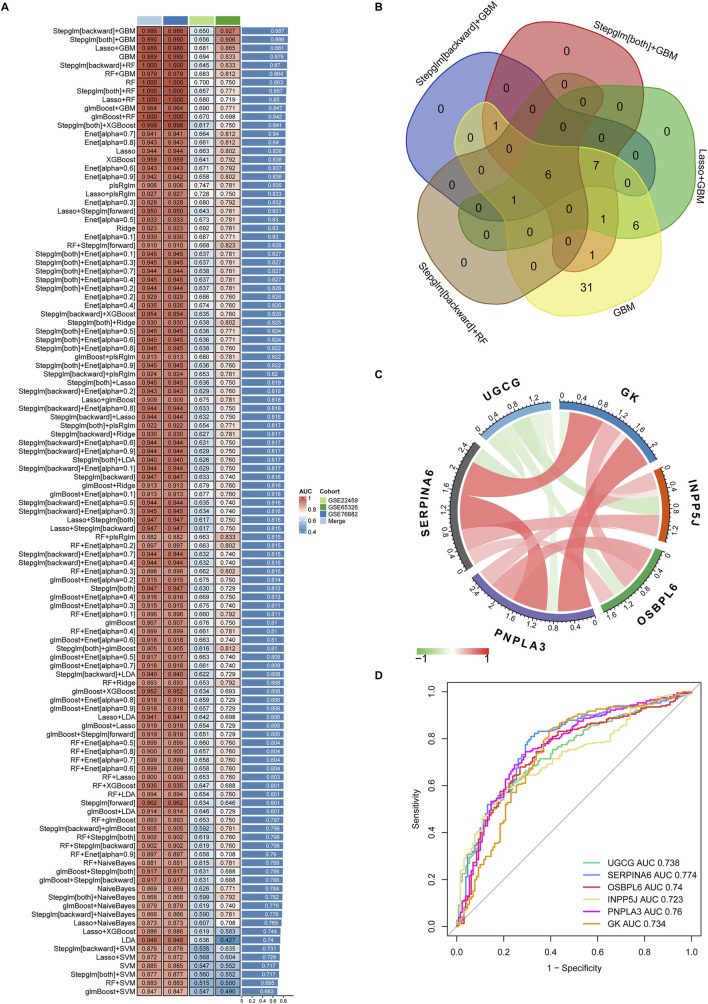
Identification and validation of key genes from 54 LMDEGs using the machine learning-based integrative model. **(A)** A comprehensive evaluation of 113 prediction models was conducted using a 10-fold cross-validation framework and calculated AUC values for each model across all datasets. **(B)** The six key LMDEGs were identified through the application of the top five machine learning algorithms. **(C)** Positive (represented by the red line) or negative (represented by the green line) correlation between the six core genes. **(D)** ROC curves of the six key LMDEGs in predicting kidney fibrosis were plotted using the pROC package in R.

We performed a total of 113 prediction models using the GSE76882 dataset through 10-fold cross-validation, calculating AUC value for each model across all validations, including GSE22459, GSE65326 and merged dataset. These combined models were then ranked across all datasets according to their AUC values, from highest to lowest (Figure 6A). Based on this ranking, the top five combined machine learning models selected for subsequent key LMDEGs screening were Stepglm [backward]+GBM, Stepglm [both]+GBM, LASSO + GBM, GBM, and Stepglm [backward]+RF (Figure 6B).

Following the application of five machine learning methods, six core LMDEGs were identified: UGCG, SERPINA6, OSBPL6, INPP5J, PNPLA3, and GK (Figure 6C). Notably, these genes exhibited significant positive and negative correlations. Specifically, UGCG demonstrated a negative correlation with the other five core LMDEGs. Apart from OSBPL6 and INPP5J, which show no correlation, the remaining LMDEGs exhibited positive correlations with each other. Subsequently, we constructed a LASSO regression model based on these core LMDEGs and evaluated their predictive performance using the ROC curve. The AUC values for each LMDEGs were as follows: UGCG (0.738), SERPINA6 (0.774), OSBPL6 (0.740), INPP5J (0.723), PNPLA3 (0.760), and GK (0.734) (Figure 6D).

### 3.4 The six core LMDEGs significantly influenced immune infiltration during the progression of KF

**FIGURE 7:**
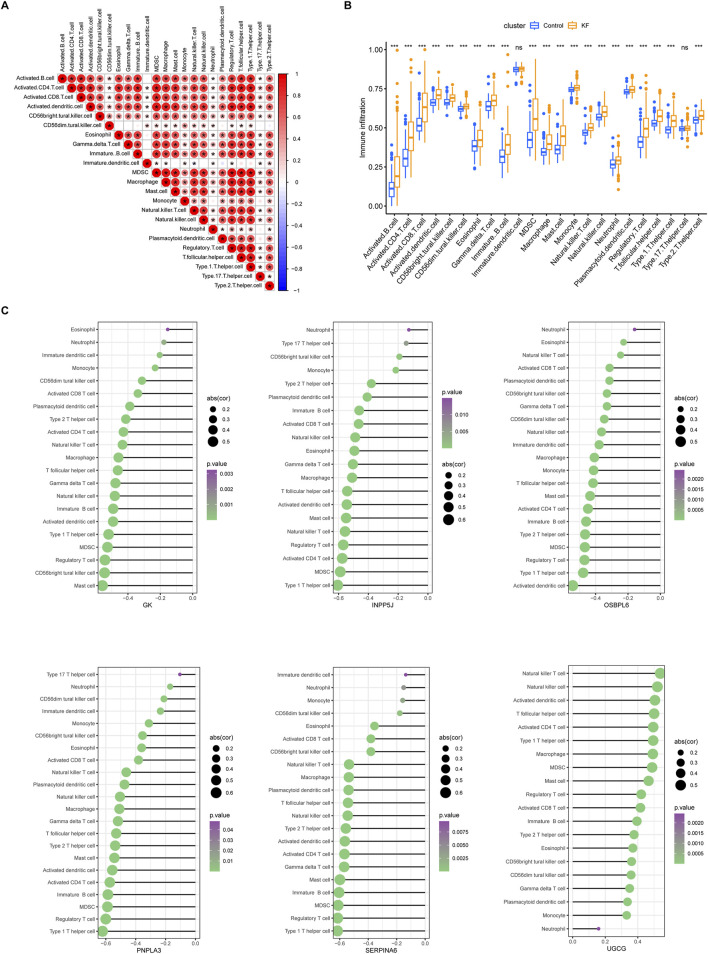
Immune infiltration analysis in ssGSEA. **(A)** A correlation heatmap displaying the proportion of immune cell infiltration within kidney tissues across all samples. **(B)** Differences in immune cell infiltration between the control group and the kidney fibrosis group. ns denotes no significant different, * denotes p < 0.05, ** denotes p < 0.01, *** denotes p < 0.001. **(C)** Six lollipop plots illustrating the distinct associations between the degree of immune infiltration and each of the key LMDEGs individually. Immunocytes with p-values below 0.05 were visualized, using bubble sizes to reflect correlation coefficients and colors to denote significance levels.

Numerous studies have demonstrated that the inflammatory response plays a critical role in the progression of KF. Consequently, we investigated immune infiltration in fibrotic kidneys and found that a diverse array of immune cells established a complex immune microenvironment during this pathological process (Figure 7A). Activated B cells exhibited strong correlations with pro-inflammatory cells such as activated CD4^+^ T cells, CD8^+^ T cells, macrophages, and mast cells. Additionally, there was a significant association between activated B cells and immune regulatory cells, including myeloid-derived suppressor cells (MDSCs), regulatory T cells, and Th1 cells. Furthermore, the infiltration of immune cells undergone distinct changes throughout the progression of KF (Figure 7B). Compared to the control group, our findings indicated that, apart from immature dendritic cells and regulatory T17 cells, all other immune cell types, including activated B cells, T cells, macrophages, and regulatory T cells, were markedly upregulated during the progression of KF. More importantly, apart from UGCG, which exhibited a consistently positive correlation with various immune cells, the other five core LMDEGs demonstrated a consistently negative correlation with these cells (Figure 7C). Specifically, Type 1 T helper cells were most closely associated with INPP5J, PNPLA3, and SERPINA6, while GK showed the strongest association with mast cells, OSBPL6 with activated dendritic cells, and UGCG with natural killer T cells. In summary, the immune cells that are closely related to KF were also significantly associated with the LMDEGs. These findings suggested that both immune infiltration and the selected lipid metabolism-related differential genes play crucial roles in the progression of KF, influencing changes in the proportions of immune cells.

### 3.5 The phenotyping capacity of the six core LMDEGs in KF

**FIGURE 8:**
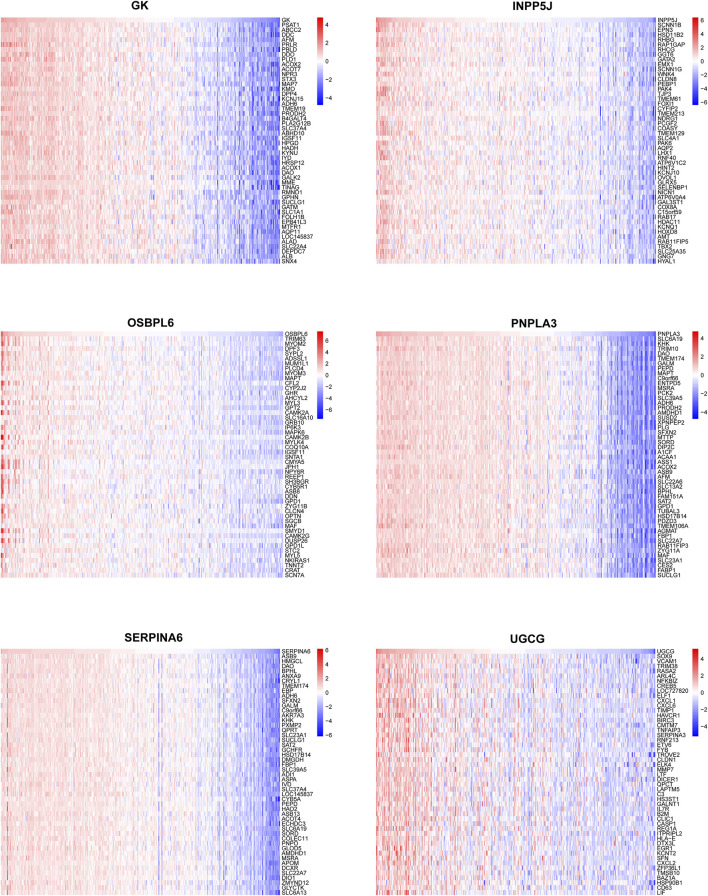
Correlation analysis between the six key LMDEGs and genomic variants. Correlation heatmaps depicting the associations between a single key LMDEG and the top 50 related genes.

**FIGURE 9:**
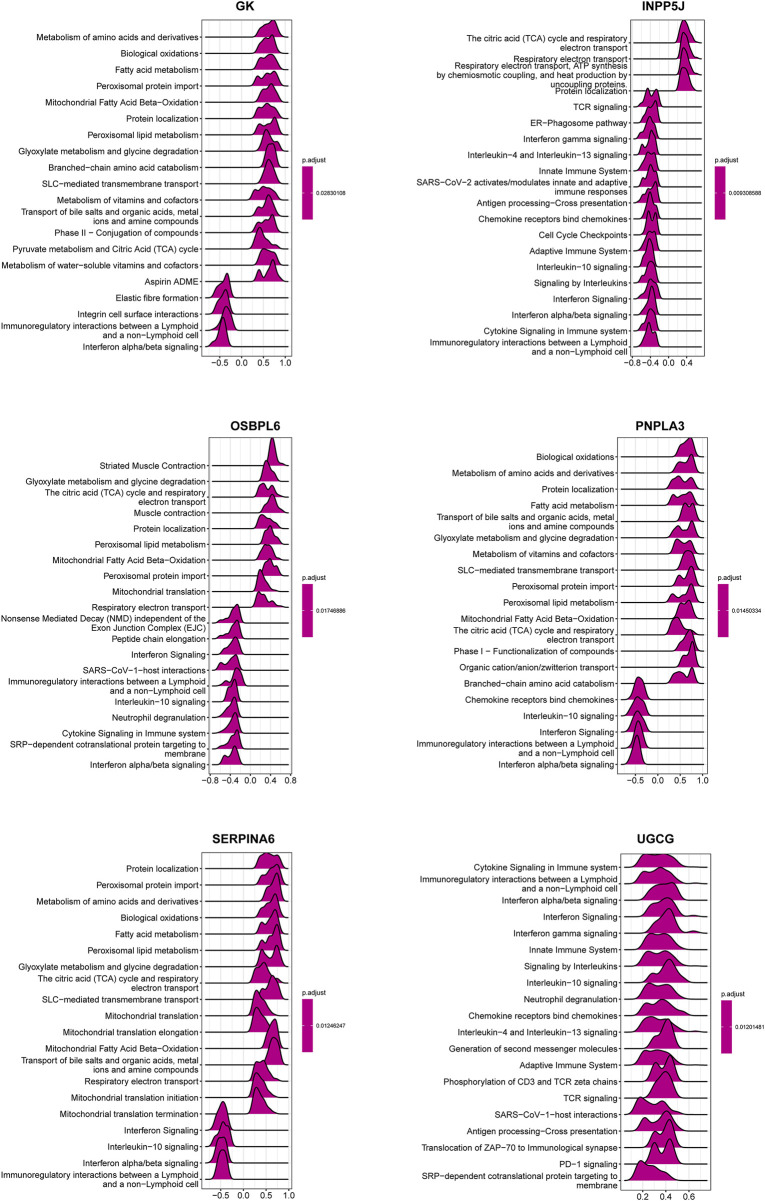
GSEA analysis of the six key LMDEGs for Reactome pathway enrichment. The ridge plots illustrating the top 20 pathways enriched in the lipid metabolism-related DEGs. Numbers on the X-axis are enrichment scores, where a value greater than zero indicates a positive correlation between a gene and a pathway, while a value less than zero indicates a negative correlation. The color indicates the corresponding p-value.

In order to catalog kidney fibrosis with core LMDEGs, we screened genes notably related to each core LMDEG (Figure 8), which were sequentially subjected to GSEA (Figure 9). Per the aforementioned results that these LMDEGs were linked to immune infiltration in KF (Figure 7), genes screened by their relationship with core LMDEGs were enriched in pathways involving energy metabolism and immune responses including fatty acid metabolism, the citric acid (TCA) cycle and respiratory electron transport, peroxisomal lipid metabolism, protein localization, interferon alpha/beta signaling and immunoregulatory interactions between a lymphoid and a non−lymphoid cell. It is important to highlight that UGCG exhibited a significant positive correlation exclusively with immune-related signaling pathways, whereas the other five core LMDEGs demonstrated notable negative correlations with these pathways. Specifically, the interferon alpha/beta signaling and immunoregulatory interactions between lymphoid and non-lymphoid cells pathways were particularly affected. Consequently, GK, OSBPL6, INPP5J, PNPLA3, and SERPINA6 played crucial roles in regulating lipid metabolic responses during KF progression. Meanwhile, UGCG, along with the aforementioned five genes, orchestrated the intricate immune microenvironment involved in this process.

**FIGURE 10:**
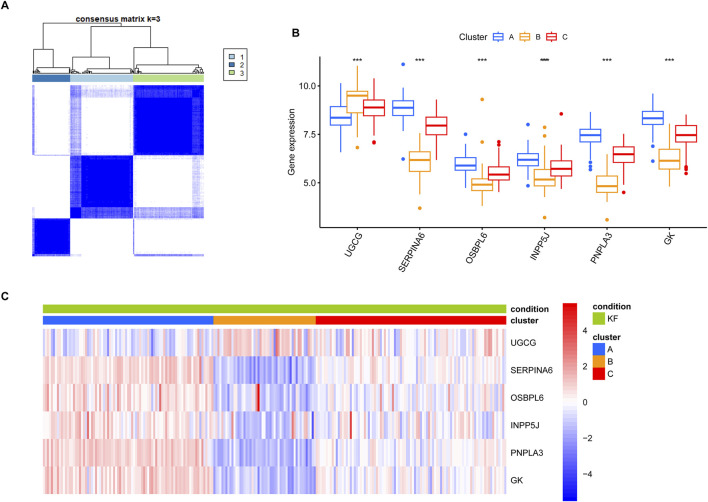
Unsupervised clustering of kidney fibrosis samples was performed based on the six key LMDEGs. **(A)** Consensus clustering analysis of kidney fibrosis samples based on profiles of LMDEGs. **(B)** A box plot illustrating the differential expression levels of the LMDEGs across the three clusters. *** denotes p < 0.001. **(C)** A heatmap showing the associations between the LMDEGs and the three clusters. The gradient transitioning from red to blue represents enrichment scores.

**FIGURE 11:**
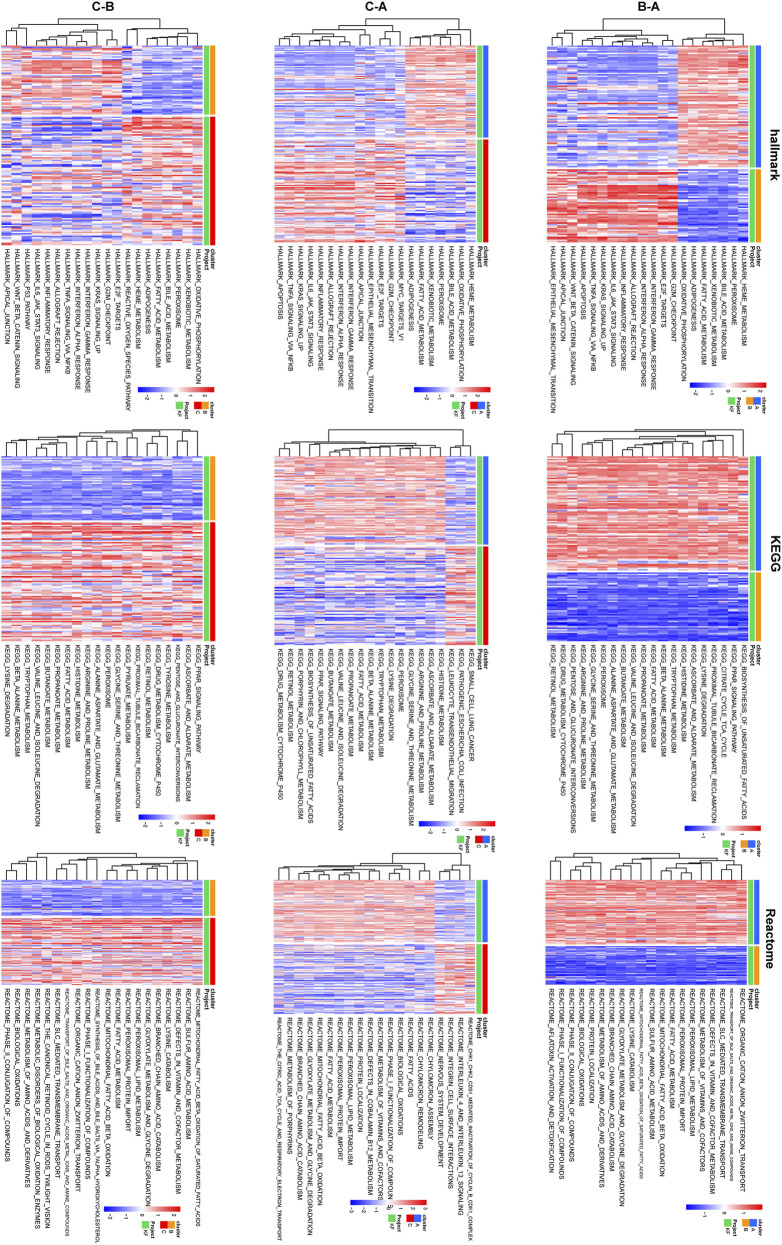
Differences in pathway enrichment among pairwise comparisons of the three clusters were shown. Heatmaps depicting the enrichment results of Hallmark, KEGG, and Reactome pathways, with each row of three heatmaps corresponding to a pair of clusters above. A gradient transitioning from red to blue represents enrichment scores.

To further investigated the convergence of KF samples in the context of the six core LMDEGs, we conducted unsupervised clustering on 231 kidney fibrosis samples obtained from three databases (Figure 10A). Specifically, Cluster A was characterized by the lowest expression level of UGCG and the highest expression levels of the remaining five genes. Conversely, Cluster B exhibited opposite expression patterns compared to Cluster A, while Cluster C displayed intermediate expression levels for all six genes (Figures 10B,C). Subsequently, GSVA pathway analysis was performed (Figure 11). The results indicated that Clusters A and B were predominantly enriched in lipid metabolism pathways, including FATTY_ACID_METABOLISM and PEROXISOMAL_LIPID_METABOLISM, with minor enrichment in immune-related pathways. In contrast, Clusters B and C were primarily enriched in immune-related pathways such as INTERFERON_GAMMA_RESPONSE and INFLAMMATORY_RESPONSE. These findings suggested that fibrotic kidneys exhibit both distinct and overlapping characteristics, and classification into three clusters may facilitated the differentiation of different phenotypes.

### 3.6 Upstream regulators influencing the expression of the six core LMDEGs

**FIGURE 12:**
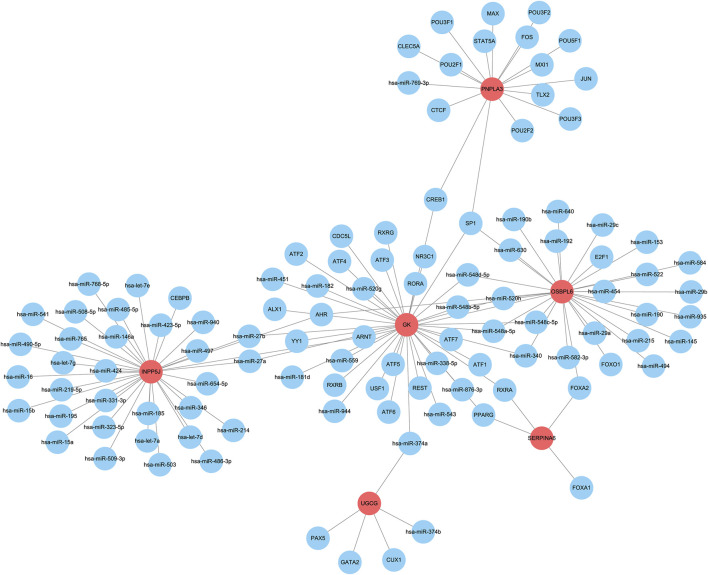
The upstream regulatory network of the six key LMDEGs. The regulatory network constructed includes the LMDEGs (represented by red bubbles) and their predicted upstream regulators (represented by blue bubbles).

To elucidate the upstream regulatory molecules of the six core LMDEGs, we constructed a regulatory network using the RegNetwork database (https://regnetworkweb.org/) and Cytoscape software (Figure 12). A total of 106 regulatory molecules were identified, with miRNAs comprising more than half of these regulators. GK was found to be regulated by the largest number of molecules, whereas SERPINA6 had the fewest regulators, specifically FOXA1, FOXA2, PPARG, and RXRA transcription factors. Additionally, several common transcription factors were observed among the core LMDEGs, including SP1, AHR, ARNT, CREB1, FOXA2, hsa-miR-27a, hsa-miR-27b, hsa-miR-340, hsa-miR-520h, hsa-miR-548a-5p, hsa-miR-548b-5p, hsa-miR-548c-5p, and hsa-miR-548d-5p. Therefore, the core LMDEGs sharing common transcription factors may exerted synergistic or antagonistic effects on the progression of KF.

## 4 Discussion

The incidence of CKD and the overall progression rate to ESRD have been rising annually (Romagnani et al., 2025). Tissue fibrosis, a common and critical pathological pathway leading to ESRD, represents an ideal focus for clinical and scientific research teams aiming to address the challenges of delaying or reversing its progression (Miguel et al., 2024). To date, current treatments primarily aim to slow the progression of KF, manage the underlying disease and associated complications (Ruiz-Ortega et al., 2022), while no specific drug has been developed to reverse this process (Chen et al., 2019). Research has demonstrated that fatty acid oxidation, which generates over three times the energy produced by aerobic glucose metabolism, serves as the predominant energy-obtaining pathway for renal tubules (Noels et al., 2021; Mitrofanova et al., 2023). However, in CKD, fatty acid oxidation is reduced, resulting in diminished energy acquisition, fat accumulation, and subsequent infiltration of inflammatory cells along with oxidative damage (Lee et al., 2024; Afshinnia et al., 2018). Therefore, fatty acid metabolic disorders are crucial in the progression of KF, and targeted modulation of lipid metabolism holds promise as a novel strategy for reversing this condition. A deeper investigation into its underlying mechanisms will not only provide a robust theoretical foundation for the development of specific drugs but also facilitate breakthroughs in clinical treatment.

However, there are currently no well-defined targets or established therapeutic mechanisms for KF in the context of lipid metabolism (Chen et al., 2022; Wei et al., 2023). Consequently, we performed a bioinformatics analysis to investigate the correlation between lipid metabolism and KF. The analysis revealed that in KF tissues, a total of 54 LMDEGs exhibited differential expression, including 14 upregulated genes such as UGCG and 40 downregulated genes such as INPP5J. Further investigation demonstrated that these genes are closely associated with fatty acid oxidation, lipid synthesis, and immune-inflammatory responses. Their aberrant expression not only disrupts energy metabolism but also facilitates immune cell infiltration, thereby exacerbating the progression of KF. Using the top five machine-learning algorithms, the six core LMDEGs were identified. Subsequent ROC analysis of these genes yielded AUC values exceeding 0.7, indicating high diagnostic and predictive accuracy.

Lipid metabolism disorders influence multiple critical pathways in KF, including extracellular matrix deposition and inflammatory responses (Qu and Jiao, 2023), sharing analogous mechanisms with fibrotic diseases such as diabetic nephropathy and hypertensive nephropathy (Kurano et al., 2023; Tanaka et al., 2022). A total of six core LMDEGs were identified: UGCG, SERPINA6, OSBPL6, INPP5J, PNPLA3, and GK. These genes exhibit close associations with the infiltration of immune cells, such as mast cells, activated dendritic cells, type 1 T helper cells, and natural killer T cells, and participate in the activation of immune signaling pathways, including interferon alpha/beta signaling, immunoregulatory interactions between lymphoid and non-lymphoid cells, and cytokine signaling in the immune system. This suggests that abnormal expression of these core LMDEGs promote the infiltration of inflammatory cells and extracellular matrix deposition, thereby accelerating the progression of KF. Literature supports that similar mechanisms are observed in other fibrotic diseases, such as diabetic nephropathy, hepatofibrosis (Horn and Tacke, 2024; Zhong et al., 2024). Notably, five core genes—SERPINA6, OSBPL6, INPP5J, PNPLA3, and GK—are negatively correlated with cells and pathways involved in the inflammatory response, whereas UGCG demonstrates a positive correlation trend, implying its potentially complex role in modulating the inflammatory response. Considering inflammatory responses, we hypothesize that UGCG exerts antagonistic effects or forms feedback regulatory mechanisms with one or more of the other five inflammatory suppressor molecules, which contributes to the worsening of inflammatory reactions during KF progression.

SERPINA6 is the gene encoding corticosteroid-binding globulin (CBG), which plays a crucial role in maintaining corticosteroid homeostasis in the plasma. Research indicates that CBG influences the negative feedback regulation of glucocorticoid receptors by modulating corticosterone availability in the adrenal glands of rodents. This function supports the normal growth, development, and functional integrity of the adrenal glands in females, highlighting its significance in sexual dimorphism (Toews et al., 2022; Yasmine Nei and rijnck, 2022). In the context of inflammation, CBG serves as a link between lipid metabolism dysregulation and inflammatory responses. Mice deficient in KLF15 exhibit a marked decrease in CBG levels, reduced plasma corticosteroid binding capacity, and increased mortality under inflammatory stress conditions, such as lipopolysaccharide (LPS) exposure. These abnormalities can be fully corrected by CBG supplementation, suggesting that KLF15 primarily contributes to the regulation of plasma corticosteroid homeostasis and inflammatory responses through its modulation of CBG expression (Zhen et al., 2022). INPP5J is a critical tumor suppressor that primarily inhibits tumor cell proliferation through the regulation of cellular signaling pathways. Research has demonstrated that both the mRNA and protein expression levels of INPP5J are markedly reduced in ovarian cancer (Zhu et al., 2015), breast cancer (Toker and Rameh, 2015), and pancreatic cancer (Zhou et al., 2024). Targeting and modulating INPP5J expression have been shown to effectively suppress tumor cell growth, indicating its potential therapeutic significance in cancer treatment. Herold, C et al. performed a family-based genome-wide association analysis and meta-analysis involving approximately 3,500 individuals. Utilizing a multivariate phenotype that integrates disease status and age at onset and identified a significant association between genetic variation in OSBPL6 (rs1347297) and the risk of Alzheimer’s disease (AD). These results underscore the potential involvement of lipid metabolism in the pathogenesis of AD (Herold et al., 2016). Metabolic-associated fatty liver disease (MAFLD) is closely related to PNPLA3 gene variations. The risk of developing ESRD in MAFLD patients is more than twice that of non-MAFLD patients (Chen et al., 2023). Studies (Targher et al., 2019)have shown that children and adolescents with the PNPLA3 rs738409 G/G genotype have significantly lower eGFR than those with G/C and C/C genotypes, and their 24-h urine protein excretion also significantly increases. These changes are independent of the severity of liver disease. Therefore, PNPLA3 gene variations not only increase the risk of ESRD in patients with MAFLD, but also may promote renal lipid accumulation and fibrosis, directly participating in the process of kidney injury (Chen et al., 2023; Mantovani and Targher, 2024). In this study, the patients with renal fibrosis included may carry a genetic variant of PNPLA3, which could influence the measured AUC value associated with PNPLA3. Therefore, future experimental studies should aim to disentangle the effect of the PNPLA3 variant and clarify its interaction with KF. UGCG is one of the key molecules involved in metabolic remodeling and has become a key therapeutic target in tumors and cardiovascular diseases, driving clinical drug development. After B-cell receptor (BCR) activation, UGCG expression is promoted. UGCG catalyzes the conversion of pro-apoptotic ceramide to anti-apoptotic glucosylceramide, which mediates chemotherapy resistance in chronic lymphocytic leukemia (CLL) (Schwamb et al., 2012). Moreover, in highly malignant melanoma, studies have shown that UGCG significantly inhibits tumor cell apoptosis induced by autophagy-lysosome inhibitors by increasing the level of sphingolipids (Jain et al., 2023). Notably, the absence of UGCG leads to the impairment of β1-adrenergic receptor (β1-AR) endocytosis in cardiomyocytes and disrupts retrograde transport from the endoplasmic reticulum to lysosomes in transgenic mice. These defects result in impaired β-adrenergic signal transduction, decreased myocardial contractility, and ultimately the development of dilated cardiomyopathy and premature death (Andersson et al., 2021). There is evidence suggesting that the expression of UGCG is upregulated in diabetic nephropathy and is associated with the downregulation of kidney-protecting genes. Especially in the condition where Fabry disease and diabetes coexist, the high expression of UGCG may lead to more severe kidney phenotypes. This indicates that UGCG may also play a certain role in kidney fibrosis, but the specific mechanism still requires further study (Sanchez-Niño et al., 2023).

In addition, within the interaction network, UGCG exhibits significant interactions with transcription factors such as PAX5, GATA2, and CUX1, as well as miRNAs including miR-374a and miR-374b. Notably, miR-374b regulates both UGCG and GK simultaneously. It may upregulate one gene (e.g., by promoting its expression) while downregulating the other (e.g., by inhibiting its expression), thereby demonstrating its bidirectional regulatory effects on UGCG and GK. This dual regulation disrupts lipid homeostasis in patients with CKD, either by reducing fatty acid oxidation or accelerating lipid synthesis, which subsequently activates inflammatory responses, promotes extracellular matrix deposition, and exacerbates KF. In glomerular podocyte injury (Luo et al., 2024), lipid accumulation leads to cytoskeleton rearrangement, insulin resistance, mitochondrial oxidative stress, and inflammatory activation. These processes, in turn, promote glomerular sclerosis and fibrosis. The Klotho gene exerts a protective effect on chronic kidney disease by reducing inflammatory responses and improving lipid metabolism (Liu et al., 2024). However, during the progression of CKD to KF, lipid metabolism disorders and inflammatory responses do not have a simple cause-and-effect relationship. Instead, they may present a cyclic relationship, where the dominant role—either lipid metabolism disorder or inflammatory response—alternates at different stages of disease progression and progresses in a synergistic manner. This understanding offers a new direction for the clinical management of chronic kidney disease. Early intervention targeting these core LMDEGs and their regulatory networks could potentially inhibit or delay the fibrotic process, offering novel therapeutic targets for the management of CKD.

From the results of GO and KEGG enrichment analysis of DEGs, we identified multiple biological signaling pathways that are closely associated with CKD and KF. These include cytokine-mediated signaling pathways, collagen-containing extracellular matrices, leukocyte-mediated immunity, and regulation of cell-cell adhesion. These findings are consistent with the current understanding of the pathogenesis of KF (Jia et al., 2025). Notably, pathways such as Phagosome and vesicle lumen suggest that intercellular signal interactions may play a pivotal role in the progression of renal fibrosis, warranting further in-depth investigation (Eirin et al., 2017; Liu et al., 2020). Moreover, the GSEA results demonstrated that DEGs were significantly enriched in pathways associated with immune infiltration, including the T cell activation pathway and B cell receptor signaling pathway. Furthermore, all six core LMDEGs exhibited significant correlations with immune cell infiltration. Specifically, UGCG was primarily enriched in natural killer T cells and natural killer cells, showing a positive correlation with immune signaling pathways such as cytokine signaling in immune system and interferon Alpha/Beta signaling. This suggests that UGCG plays a pro-inflammatory role in the progression of KF. The remaining five core LMDEGs (SERPINA6, OSBPL6, INPP5J, PNPLA3, and GK) displayed negative correlations with immune signaling pathways, such as interferon Alpha/Beta signaling, while being positively correlated with lipid metabolism pathways, including the citric acid (TCA) cycle and respiratory electron transport. These findings indicate that these core LMDEGs may contribute to promoting fatty acid metabolism, correcting lipid metabolic disorders, inhibiting inflammatory cells, and counteracting immune infiltration reactions during KF. Through the analysis of the aforementioned results, we have drawn the conclusion that immune infiltration is intricately involved in the entire progression of KF. Furthermore, genes associated with lipid metabolism exert regulatory effects on immune infiltration (He et al., 2024), indicating a potential correlation between immune infiltration and lipid metabolic disorders, both of which collectively influence the process of KF. This area will constitute one of our key research focuses in the future. Additionally, we have observed certain conclusions from other studies that are inconsistent with our findings. For instance, some pathways were not significantly enriched in our study, which may be attributable to variations in sample selection, experimental conditions, or analytical methodologies and thus warrant further investigation and consideration.

The unsupervised clustering analysis identified three distinct molecular subtypes of KF, each with potential therapeutic implications. Cluster B, marked by elevated UGCG expression and activation of immune-related pathways, may represent an “inflammatory” subtype that could potentially benefit from immunomodulatory therapies. In contrast, the lipid metabolism-dominant signature of Cluster A suggests a possible responsiveness to PPAR agonists or other lipid-modulating agents. The intermediate phenotype of Cluster C might necessitate combination therapies targeting both metabolic and inflammatory pathways. These findings are consistent with the emerging paradigm of precision medicine in KF, where molecular subtyping could inform therapy selection. However, the clinical significance of these subtypes remains unclear without longitudinal data linking them to disease progression or treatment response. Furthermore, the underlying biological mechanisms driving these subtype distinctions warrant further investigation, particularly to determine whether they reflect distinct disease etiologies or different stages along a common pathogenic continuum.

Despite the robust bioinformatics methodologies utilized in this study, several limitations must be acknowledged. First, the findings lack experimental validation via functional assays such as gene knockout or overexpression models, which are essential for establishing causal relationships between the identified LMDEGs (particularly UGCG and SERPINA6) and fibrotic progression. Second, although the machine learning models exhibited strong predictive performance (AUC 0.723–0.774), the relatively small sizes of the validation cohorts (GSE22459: n = 65; GSE65326: n = 22) and potential batch effects, even after sva/limma correction, may constrain their generalizability. Third, the absence of longitudinal clinical data prevents an evaluation of whether the three molecular subtypes demonstrate differential prognostic outcomes or therapeutic responses. Future studies should integrate single-cell RNA sequencing to elucidate immune-metabolic crosstalk at a cellular level and validate subtype-specific treatment strategies in preclinical models.

## 5 Conclusion

This study identifies the dysregulation of the lipid metabolism-immune network as a hallmark feature of KF, with UGCG functioning as a central pro-inflammatory hub. Furthermore, genes such as SERPINA6 and OSBPL6, along with other LMDEGs, play modulatory roles in lipid metabolic pathways. The six-gene signature derived from machine learning not only stratifies patients into clinically relevant subtypes—characterized by lipid-dominant versus immune-dominant phenotypes—but also reveals shared transcriptional regulators, including PPAR and the miR-27 family, which may coordinately drive fibrotic progression. These findings provide a foundational framework for the development of precision therapeutics targeting specific components of the LMDEG-immune axis. However, translational applications will require validation in larger, prospectively collected cohorts with matched histopathological and functional data.

## Data Availability

The original contributions presented in the study are included in the article/Supplementary Material, further inquiries can be directed to the corresponding authors.

## Glossary

KF: kidney fibrosis
CKD: chronic kidney disease
eGFR: estimated glomerular filtration rate
ACR: albumin-to-creatinine ratio
ESRD: end-stage renal disease
DEGs: the differentially expressed genes
LMDEGs: lipid metabolism-associated DEGs
GO: Gene Ontology
GEO: Gene Expression Omnibus
KEGG: Kyoto Encyclopedia of Genes and Genomes
LASSO: Least Absolute Shrinkage and Selection Operator
ssGSEA: lingle-sample Gene Set Enrichment Analysis
GSVA: Gene Set Variation Analysis
PCA: Principal Component Analysis
LIMMA: Linear Models for Microarray Data
RSF: Random Survival Forest
GBM: Gradient Boosting Machine
Survival-SVM: Survival Support Vector Machine
SuperPC: Supervised Principal Components Analysis
plsRcox: Partial Least Squares Regression for Cox models
Enet: Elastic Net
AUC: area under the curve
ROC: receiver operating characteristic
BP: biological process
MF: molecular function
CC: cellular component
FDR: false discovery rate
MDSCs: myeloid-derived suppressor cells
CBG: corticosteroid-binding globulin
AD: Alzheimer’s disease
MAFLD: metabolic-associated fatty liver disease
BCR: b-cell receptor
CLL: chronic lymphocytic leukemia
β1-AR: β1-adrenergic receptor

## References

[B1] Afshinnia F. Rajendiran T. M. Soni T. Byun J. Wernisch S. Sas K. M. (2018). Impaired β-oxidation and altered complex lipid fatty acid partitioning with advancing CKD. J. Am. Soc. Nephrol. 29, 295–306. 10.1681/ASN.2017030350 29021384 PMC5748913

[B2] Andersson L. Cinato M. Mardani I. Miljanovic A. Arif M. Koh A. (2021). Glucosylceramide synthase deficiency in the heart compromises β1-adrenergic receptor trafficking. Eur. Heart J. 42, 4481–4492. 10.1093/eurheartj/ehab412 34297830 PMC8599074

[B3] Chen T. K. Knicely D. H. Grams M. E. (2019). Chronic kidney disease diagnosis and management: a review. JAMA 322, 1294–1304. 10.1001/jama.2019.14745 31573641 PMC7015670

[B4] Chen Y. Yan Q. Lv M. Song K. Dai Y. Huang Y. (2020). Involvement of FATP2-mediated tubular lipid metabolic reprogramming in renal fibrogenesis. Cell Death Dis. 11, 994. 10.1038/s41419-020-03199-x 33219209 PMC7679409

[B5] Chen Y. Y. Chen X. G. Zhang S. (2022). Druggability of lipid metabolism modulation against renal fibrosis. Acta Pharmacol. Sin. 43, 505–519. 10.1038/s41401-021-00660-1 33990764 PMC8888625

[B6] Chen S. Pang J. Huang R. Xue H. Chen X. (2023). Association of MAFLD with end-stage kidney disease: a prospective study of 337,783 UK Biobank participants. Hepatol. Int. 17, 595–605. 10.1007/s12072-023-10486-0 36809487

[B7] Eirin A. Zhu X.-Y. Puranik A. S. Tang H. McGurren K. A. van Wijnen A. J. (2017). Mesenchymal stem cell–derived extracellular vesicles attenuate kidney inflammation. Kidney Int. 92, 114–124. 10.1016/j.kint.2016.12.023 28242034 PMC5483390

[B8] Feinberg A. P. (2018). The key role of epigenetics in human disease prevention and mitigation. N. Engl. J. Med. 378, 1323–1334. 10.1056/NEJMra1402513 29617578 PMC11567374

[B9] Foreman K. J. Marquez N. Dolgert A. Fukutaki K. Fullman N. McGaughey M. (2018). Forecasting life expectancy, years of life lost, and all-cause and cause-specific mortality for 250 causes of death: reference and alternative scenarios for 2016-40 for 195 countries and territories. Lancet 392, 2052–2090. 10.1016/S0140-6736(18)31694-5 30340847 PMC6227505

[B10] Francis A. Harhay M. N. Ong A. C. M. Tummalapalli S. L. Ortiz A. Fogo A. B. (2024). Chronic kidney disease and the global public health agenda: an international consensus. Nat. Rev. Nephrol. 20, 473–485. 10.1038/s41581-024-00820-6 38570631

[B11] GBD Chronic Kidney Disease Collaboration (2020). Global, regional, and national burden of chronic kidney disease, 1990-2017: a systematic analysis for the Global Burden of Disease Study 2017. Lancet 395, 709–733. 10.1016/S0140-6736(20)30045-3 32061315 PMC7049905

[B12] He L. Ye Q. Zhu Y. Zhong W. Xu G. Wang L. (2024). Lipid metabolism‐related gene signature predicts prognosis and indicates immune microenvironment infiltration in advanced gastric cancer. Gastroenterology Res. Pract. 2024, 6639205. 10.1155/2024/6639205 38440405 PMC10911888

[B13] Herold C. Hooli B. V. Mullin K. Liu T. Roehr J. T. Mattheisen M. (2016). Family-based association analyses of imputed genotypes reveal genome-wide significant association of Alzheimer’s disease with OSBPL6, PTPRG, and PDCL3. Mol. Psychiatry 21, 1608–1612. 10.1038/mp.2015.218 26830138 PMC4970971

[B14] Horn P. Tacke F. (2024). Metabolic reprogramming in liver fibrosis. Cell Metab. 36, 1439–1455. 10.1016/j.cmet.2024.05.003 38823393

[B15] Huang R. Fu P. Ma L. (2023). Kidney fibrosis: from mechanisms to therapeutic medicines. Signal Transduct. Target Ther. 8, 129. 10.1038/s41392-023-01379-7 36932062 PMC10023808

[B16] Jadoul M. Aoun M. Masimango Imani M. (2024). The major global burden of chronic kidney disease. Lancet Glob. Health 12, e342–e343. 10.1016/s2214-109x(24)00050-0 38365398

[B17] Jain V. Harper S. L. Versace A. M. Fingerman D. Brown G. S. Bhardwaj M. (2023). Targeting UGCG overcomes resistance to lysosomal autophagy inhibition. Cancer Discov. 13, 454–473. 10.1158/2159-8290.CD-22-0535 36331284 PMC9905280

[B18] Jia H. Yue G. Li P. Peng R. Ruyue J. Yuhan C. (2025). Neutrophil extracellular traps license macrophage production of chemokines to facilitate CD8+ T cell infiltration in obstruction-induced renal fibrosis. Protein Cell, pwaf020. 10.1093/procel/pwaf020 39998389

[B19] Kuppe C. Ibrahim M. M. Kranz J. Zhang X. Ziegler S. Perales-Paton J. (2021). Decoding myofibroblast origins in human kidney fibrosis. Nature 589, 281–286. 10.1038/s41586-020-2941-1 33176333 PMC7611626

[B20] Kurano M. Tsukamoto K. Shimizu T. Hara M. Yatomi Y. (2023). Apolipoprotein M/sphingosine 1-phosphate protects against diabetic nephropathy. Transl. Res. 258, 16–34. 10.1016/j.trsl.2023.02.004 36805561

[B21] Lee L. E. Doke T. Mukhi D. Susztak K. (2024). The key role of altered tubule cell lipid metabolism in kidney disease development. Kidney Int. 106, 24–34. 10.1016/j.kint.2024.02.025 38614389 PMC11193624

[B22] Li L. Fu H. Liu Y. (2022). The fibrogenic niche in kidney fibrosis: components and mechanisms. Nat. Rev. Nephrol. 18, 545–557. 10.1038/s41581-022-00590-z 35788561

[B23] Li X. Chen J. Li J. Zhang Y. Xia J. Du H. (2025). ATGL regulates renal fibrosis by reprogramming lipid metabolism during the transition from AKI to CKD. Mol. Ther. 33, 805–822. 10.1016/j.ymthe.2024.12.053 39748508 PMC11853023

[B24] Liu X. Miao J. Wang C. Zhou S. Chen S. Ren Q. (2020). Tubule-derived exosomes play a central role in fibroblast activation and kidney fibrosis. Kidney Int. 97, 1181–1195. 10.1016/j.kint.2019.11.026 32139089

[B25] Liu J. Wang H. Liu Q. Long S. Wu Y. Wang N. (2024). Klotho exerts protection in chronic kidney disease associated with regulating inflammatory response and lipid metabolism. Cell Biosci. 14, 46–29. 10.1186/s13578-024-01226-4 38584258 PMC11000353

[B26] Luo Z. Chen Z. Hu J. Ding G. (2024). Interplay of lipid metabolism and inflammation in podocyte injury. Metabolism 150, 155718. 10.1016/j.metabol.2023.155718 37925142

[B27] Mantovani A. Targher G. (2024). *PNPLA3* variation and kidney disease. Liver Int. 45, e16010. 10.1111/liv.16010 38873992

[B28] Miguel V. Shaw I. W. Kramann R. (2024). Metabolism at the crossroads of inflammation and fibrosis in chronic kidney disease. Nat. Rev. Nephrol. 21, 39–56. 10.1038/s41581-024-00889-z 39289568

[B29] Mitrofanova A. Merscher S. Fornoni A. (2023). Kidney lipid dysmetabolism and lipid droplet accumulation in chronic kidney disease. Nat. Rev. Nephrol. 19, 629–645. 10.1038/s41581-023-00741-w 37500941 PMC12926870

[B30] Modena B. D. Kurian S. M. Gaber L. W. Waalen J. Su A. I. Gelbart T. (2016). Gene expression in biopsies of acute rejection and interstitial fibrosis/tubular atrophy reveals highly shared mechanisms that correlate with worse long‐term outcomes. Am. J. Transplant. 16, 1982–1998. 10.1111/ajt.13728 26990570 PMC5501990

[B31] Noels H. Lehrke M. Vanholder R. Jankowski J. (2021). Lipoproteins and fatty acids in chronic kidney disease: molecular and metabolic alterations. Nat. Rev. Nephrol. 17, 528–542. 10.1038/s41581-021-00423-5 33972752

[B32] Park W. D. Griffin M. D. Cornell L. D. Cosio F. G. Stegall M. D. (2010). Fibrosis with inflammation at one year predicts transplant functional decline. J. Am. Soc. Nephrol. 21, 1987–1997. 10.1681/ASN.2010010049 20813870 PMC3014013

[B33] Qu L. Jiao B. (2023). The interplay between immune and metabolic pathways in kidney disease. Cells 12, 1584. 10.3390/cells12121584 37371054 PMC10296595

[B34] Romagnani P. Agarwal R. Chan J. C. N. Levin A. Kalyesubula R. Karam S. (2025). Chronic kidney disease. Nat. Rev. Dis. Prim. 11, 8. 10.1038/s41572-024-00589-9 39885176

[B35] Ruiz-Ortega M. Rayego-Mateos S. Lamas S. Ortiz A. Rodrigues-Diez R. R. (2020). Targeting the progression of chronic kidney disease. Nat. Rev. Nephrol. 16, 269–288. 10.1038/s41581-019-0248-y 32060481

[B36] Ruiz-Ortega M. Lamas S. Ortiz A. (2022). Antifibrotic agents for the management of CKD: a review. Am. J. Kidney Dis. 80, 251–263. 10.1053/j.ajkd.2021.11.010 34999158

[B37] Sakers A. De Siqueira M. K. Seale P. Villanueva C. J. (2022). Adipose-tissue plasticity in health and disease. Cell 185, 419–446. 10.1016/j.cell.2021.12.016 35120662 PMC11152570

[B38] Sanchez-Niño M. D. Ceballos M. I. Carriazo S. Pintor-Chocano A. Sanz A. B. Saleem M. (2023). Interaction of Fabry disease and diabetes mellitus: suboptimal recruitment of kidney protective factors. Int. J. Mol. Sci. 24, 1–14. 10.3390/ijms242115853 37958836 PMC10650640

[B39] Schwamb J. Feldhaus V. Baumann M. Patz M. Brodesser S. Brinker R. (2012). B-cell receptor triggers drug sensitivity of primary CLL cells by controlling glucosylation of ceramides. Blood 120, 3978–3985. 10.1182/blood-2012-05-431783 22927247

[B40] Scorletti E. Carr R. M. (2022). A new perspective on NAFLD: focusing on lipid droplets. J. Hepatol. 76, 934–945. 10.1016/j.jhep.2021.11.009 34793866

[B41] Tanaka S. Zheng S. Kharel Y. Fritzemeier R. G. Huang T. Foster D. (2022). Sphingosine 1-phosphate signaling in perivascular cells enhaces inflammation and fibrosis in the kidney. Sci. Transl. Med. 14, 1–15. 10.1126/scitranslmed.abj2681 PMC9873476 35976996

[B42] Targher G. Mantovani A. Alisi A. Mosca A. Panera N. Byrne C. D. (2019). Relationship between PNPLA3 rs738409 polymorphism and decreased kidney function in children with NAFLD. Hepatology 70, 142–153. 10.1002/hep.30625 30912854

[B43] Terry A. R. Hay N. (2024). Emerging targets in lipid metabolism for cancer therapy. Trends Pharmacol. Sci. 45, 537–551. 10.1016/j.tips.2024.04.007 38762377 PMC11162322

[B44] Toews J. N. C. Philippe T. J. Hill L. A. Dordevic M. Miguelez-Crespo A. Homer N. Z. M. (2022). Corticosteroid-binding globulin (SERPINA6) establishes postpubertal sex differences in rat adrenal development. Endocrinology 163, bqac152. 10.1210/endocr/bqac152 36112420

[B45] Toker A. Rameh L. (2015). PIPPing on AKT1: how many phosphatases does it take to turn off PI3K? Cancer Cell 28, 143–145. 10.1016/j.ccell.2015.07.010 26267528

[B46] Toki D. Zhang W. Hor K. L. M. Liuwantara D. Alexander S. I. Yi Z. (2014). The role of macrophages in the development of human renal allograft fibrosis in the First year after transplantation. Am. J. Transplant. 14, 2126–2136. 10.1111/ajt.12803 25307039

[B47] Wei X. Hou Y. Long M. Jiang L. Du Y. (2023). Advances in energy metabolism in renal fibrosis. Life Sci. 312, 121033. 10.1016/j.lfs.2022.121033 36270427

[B48] Yasmine Neirijnck A. S. (2022). Rats, adrenals and the surprising role of the corticosteroid-binding globulin (CBG) in sexual dimorphism, 164. 10.1210/endocr/bqac190 36402142

[B49] Yuan Q. Tang B. Zhang C. (2022). Signaling pathways of chronic kidney diseases, implications for therapeutics. Signal Transduct. Target Ther. 7, 182. 10.1038/s41392-022-01036-5 35680856 PMC9184651

[B50] Zhen Z. Elsarrag S. Z. Duan Q. LaGory E. L. Wang Z. Alexanian M. (2022). KLF15 cistromes reveal a hepatocyte pathway governing plasma corticosteroid transport and systemic inflammation. Sci. Adv. 8, eabj2917. 10.1126/sciadv.abj2917 35263131 PMC8906731

[B51] Zhong D. Chen J. Qiao R. Song C. Hao C. Zou Y. (2024). Genetic or pharmacologic blockade of mPGES-2 attenuates renal lipotoxicity and diabetic kidney disease by targeting Rev-Erbα/FABP5 signaling. Cell Rep. 43, 114075. 10.1016/j.celrep.2024.114075 38583151

[B52] Zhou W. Deng X. Liu L. Yuan Y. Meng X. Ma J. (2024). PELI1 overexpression contributes to pancreatic cancer progression through upregulating ubiquitination-mediated INPP5J degradation. Cell. Signal. 120, 111194. 10.1016/j.cellsig.2024.111194 38685520

[B53] Zhu T. Yuan J. Wang Y. Gong C. Xie Y. Li H. (2015). MiR-661 contributed to cell proliferation of human ovarian cancer cells by repressing INPP5J expression. Biomed. and Pharmacother. 75, 123–128. 10.1016/j.biopha.2015.07.023 26282217

